# Pharmacometabolomics Reveals Racial Differences in Response to Atenolol Treatment

**DOI:** 10.1371/journal.pone.0057639

**Published:** 2013-03-11

**Authors:** William R. Wikoff, Reginald F. Frye, Hongjie Zhu, Yan Gong, Stephen Boyle, Erik Churchill, Rhonda M. Cooper-Dehoff, Amber L. Beitelshees, Arlene B. Chapman, Oliver Fiehn, Julie A. Johnson, Rima Kaddurah-Daouk, Pharmacometabolomics Research Network

**Affiliations:** 1 UC Davis Genome Center, University of California Davis, Davis, California, United States of America; 2 Department of Pharmacotherapy and Translational Research and Center for Pharmacogenomics, University of Florida, Gainesville, Florida, United States of America; 3 Department of Psychiatry, Duke University Medical Center, Durham, North Carolina, United States of America; 4 Department of Medicine, University of Maryland School of Medicine, Baltimore, Maryland, United States of America; 5 Department of Medicine, Emory University, Atlanta, Georgia, United States of America; 6 Duke Institute for Brain Sciences, Duke University, Durham, North Carolina, United States of America; UAE University, United Arab Emirates

## Abstract

Antihypertensive drugs are among the most commonly prescribed drugs for chronic disease worldwide. The response to antihypertensive drugs varies substantially between individuals and important factors such as race that contribute to this heterogeneity are poorly understood. In this study we use metabolomics, a global biochemical approach to investigate biochemical changes induced by the beta-adrenergic receptor blocker atenolol in Caucasians and African Americans. Plasma from individuals treated with atenolol was collected at baseline (untreated) and after a 9 week treatment period and analyzed using a GC-TOF metabolomics platform. The metabolomic signature of atenolol exposure included saturated (palmitic), monounsaturated (oleic, palmitoleic) and polyunsaturated (arachidonic, linoleic) free fatty acids, which decreased in Caucasians after treatment but were not different in African Americans (p<0.0005, q<0.03). Similarly, the ketone body 3-hydroxybutyrate was significantly decreased in Caucasians by 33% (p<0.0001, q<0.0001) but was unchanged in African Americans. The contribution of genetic variation in genes that encode lipases to the racial differences in atenolol-induced changes in fatty acids was examined. SNP rs9652472 in LIPC was found to be associated with the change in oleic acid in Caucasians (p<0.0005) but not African Americans, whereas the PLA2G4C SNP rs7250148 associated with oleic acid change in African Americans (p<0.0001) but not Caucasians. Together, these data indicate that atenolol-induced changes in the metabolome are dependent on race and genotype. This study represents a first step of a pharmacometabolomic approach to phenotype patients with hypertension and gain mechanistic insights into racial variability in changes that occur with atenolol treatment, which may influence response to the drug.

## Introduction

Hypertension is the most common chronic disease world-wide with an estimated one billion individuals affected [1]. The cause of blood pressure elevation is not known in the vast majority of patients with essential hypertension, thus it is difficult to project prognosis or predict optimal treatment in an individual patient. Although multiple antihypertensive drug classes are available for treatment of hypertension, only about 50% of individuals have adequate blood pressure (BP) lowering with any given drug therapy and less than 40% of hypertensive patients have controlled BP [2], [3]. Additionally, there are well defined differences in drug response by race but mechanisms underlying these differences in response are poorly understood.

β-blockers such as atenolol are important first line antihypertensive treatment, but not all patients respond and these drugs are associated with adverse metabolic effects, specifically adverse changes in glucose, triglycerides and uric acid, all of which can have negative cardiovascular consequences long-term [4], [5]. Unfortunately, there are no well-defined biomarkers to identify patients that will have a beneficial therapeutic response or experience the adverse metabolic consequences of these drugs.

Metabolomics is a global biochemical approach that provides powerful tools for defining perturbations in metabolic pathways and networks in human disease [6]–[10]. The metabolome defines a metabolic state as regulated by net interactions between gene and environmental influences and provides information that can possibly bridge the gap between genotype and phenotype. Targeted and non-targeted metabolomic approaches have been used to define pathways implicated in variation of response to drugs such as escitalopram [11] and simvastatin [12], [13] leading to the emergence of a new field: pharmacometabolomics [9], [14], [15]. Indeed, pharmacometabolomics can be used to define a unique signature that represents changes in the metabolome induced by drug treatment. This signature can provide insight into the mechanism of variation in drug response caused by factors including race and genetics.

In the present study, a mass spectrometry based global metabolomics approach was used to analyze effects of the β-blocker atenolol in patients participating in the Pharmacogenomic Evaluation of Antihypertensive Responses (PEAR) study [16]. PEAR is a randomized clinical trial designed to identify genetic determinants of blood pressure and adverse metabolic responses to a thiazide diuretic and β-blocker. Responses to the β_1_-selective blocker atenolol or the diuretic hydrochlorothiazide as monotherapy and then in combination were determined in 768 patients with mild to moderate hypertension. Metabolomic analyses were conducted in a subset of 272 patients receiving atenolol monotherapy. The objective of this study was to characterize the metabolomic signature of drug treatment as a first step to determine whether metabolomics might provide novel mechanistic insight into racial differences in response to β-blockers. Because the analysis showed a strong fatty acid signature that also differed by race, we tested the hypothesis that genetic variation in genes encoding lipases might be associated with observed atenolol-induced changes in different racial groups.

## Materials and Methods

### Subjects

Plasma samples and associated clinical data were collected as part of the Pharmacogenomic Evaluation of Antihypertensive Response (PEAR) study, which is a prospective, randomized, parallel group titration study undertaken in primary care patients with mild to moderate essential hypertension. The objectives and design of the PEAR study have been described previously [16]. Subjects of any self-defined race or ethnicity, aged 17–65 years, were enrolled at the University of Florida (Gainesville, FL), Emory University (Atlanta, GA), and the Mayo Clinic (Rochester, MN). The study was approved by the institutional review board at each institution, and all participants provided informed, written consent prior to being screened for study participation. Enrolled subjects had newly diagnosed, untreated or treated hypertension; if treated, the antihypertensive(s) was discontinued with a minimum washout of 18 days. Briefly, exclusion criteria included diastolic blood pressure (DBP) >110 mm Hg or systolic blood pressure (SBP) >180 mm Hg, secondary hypertension, cardiovascular disease, diabetes, renal insufficiency (serum creatinine >1.5 in male patients and >1.4 in female patients), liver enzymes >2.5 times the upper limit of normal, and treatment with BP-raising drugs, among others.

### Study protocol

Enrolled subjects were randomly assigned at each study site to receive hydrochlorothiazide or atenolol monotherapy; the focus of the metabolomics analyses reported herein is the atenolol monotherapy treatment arm. Atenolol was initiated at 50.0 mg daily for 3 weeks and titrated to 100.0 mg daily on the basis of blood pressure; treatment continued for an additional 6 weeks. Blood pressure was assessed at baseline and after 9 weeks of atenolol treatment by home-recorded blood pressure measurements using a Microlife model 3AC1-PC home BP monitor (BP Microlife, Minneapolis, MN). The device was set to measure BP in triplicate with each activation and to store the average systolic and diastolic BPs and the time of each set of measurements.

### Plasma samples

Subjects included in the metabolomics analyses (n = 272) were randomly selected from each quartile of blood pressure response, defined as the difference in BP after atenolol treatment from the BP at baseline. Subjects were balanced by race and to the extent possible by sex. Pre- and post-treatment fasting plasma samples were collected at baseline and after 9 weeks of atenolol treatment.

### GC-TOF Mass Spectrometry

The study design was entered into the SetupX database [17]. Plasma samples were aliquotted and maintained at −80°C until use, at which point 30 µl of plasma samples were thawed, extracted and derivatized [18]. Briefly, 15 µl aliquots were extracted with 1 ml of degassed acetonitrile∶isopropanol∶water (3∶3∶2) at −20°C, centrifuged and decanted with subsequent evaporation of the solvent to complete dryness. A clean-up step with acetonitrile/water (1∶1) removed membrane lipids and triglycerides and the supernatant was again dried down. Internal standards C8–C30 FAMEs were added and the sample was derivatized with methoxyamine hydrochloride in pyridine and subsequently by MSTFA (Sigma-Aldrich) for trimethylsilylation of acidic protons.

A Gerstel MPS2 automatic liner exchange system was used to inject 1 µl of sample at 50°C (ramped to 250°C) in splitless mode with a 25 sec splitless time. An Agilent 6890 gas chromatograph (Santa Clara, CA) was used with a 30 m long, 0.25 mm i.d. Rtx5Sil-MS column with 0.25 µm 5% diphenyl film; an additional 10 m integrated guard column was used (Restek, Bellefonte PA). Chromatography was performed at a constant flow of 1 ml/min, ramping the oven temperature from 50°C for to 330°C over 22 min. Mass spectrometry used a Leco Pegasus IV time of flight mass (TOF) spectrometer with 280°C transfer line temperature, electron ionization at −70 V and an ion source temperature of 250°C. Mass spectra were acquired from *m/z* 85–500 at 20 spectra/sec and 1750 V detector voltage. Result files were exported to our servers and further processed by our metabolomics BinBase database. All database entries in BinBase were matched against the Fiehn mass spectral library of 1,200 authentic metabolite spectra using retention index and mass spectrum information or the NIST05 commercial library. Identified metabolites were reported if present with at least 50% of the samples per study design group (as defined in the SetupX database). Quantitative data were normalized to the sum intensities of all known metabolites and used for statistical investigation.

### Genotyping of Lipase Candidate Genes

Genotypes of 16 lipase genes were obtained from the Illumina Human 50 K cardiovascular chip [19], a customized gene-centric array including ∼2100 genes and ∼50,000 SNPs genotyped using the Infinium II Assay (Illumina, San Diego, CA). Genotypes were called using GenomeStudio software version 2011.1 and the Genotyping Module version 1.9 calling algorithm (Illumina, San Diego, CA). Participants were excluded if sample genotype call rates were below 95% and SNPs were excluded if genotype call rates were below 90%. Sample contamination was detected by checking gender mismatches using X chromosome genotype data and cryptic relatedness was estimated by pairwise identity-by-descent (IBD) analysis implemented using PLINK [20]. After the QC procedures, the total SNP call rate in the remaining individuals was 99.799%. Hardy-Weinberg equilibrium was assessed by chi-square test with one degree of freedom. There were 463 SNPs included in the genetic association analysis.

### Data Analysis

A Wilcoxon signed rank test was used to detect metabolites that were significantly changed by drug treatment. The difference in metabolic change between two race groups, Caucasian and African American, was evaluated using a Wilcoxon rank sum test. Q-values [21] were calculated to control for multiple testing false discovery rate (FDR). Correlation matrixes were used to visualize the correlation between metabolites. The modulated modularity clustering algorithm [22] was used to cluster metabolites based on their pairwise Spearman's correlation coefficients. Pathways and networks were analyzed using multiple approaches. MetaMapp [23] was used to calculate metabolic networks, which were displayed using Cytoscape [24]. Multiple databases were used in the process of data analysis. These included KEGG [25] and PharmGKB [26].

Associations of the 463 SNPs in the lipase genes with oleic acid response to atenolol monotherapy were evaluated using linear regression, adjusting for baseline oleic acid, age, gender and the first 2 principal components for ancestry, which correspond to European and African ancestry, respectively. P values of <0.0001 (0.05/463) were considered statistically significant. Genetic association analysis was performed using PLINK [20] assuming additive mode of inheritance.

### Network Modeling

The process for constructing a model based upon metabolomics data is quite different from the process for proteomics, transcriptomics or genomics datasets. This is because concept or text-based associations (for example GO categories or MESH headings) are not associated with small molecule compounds as they are for proteins or genes. While pathway databases such as KEGG can be used to deduce some mechanisms, the available data are extremely limited. For example, only a fraction of the known human metabolome is linked to pathways, and secondary processes such as gut microbiome-generated effects [27] and much of lipid metabolism are not included. For this reason, less direct methods using existing tools must be used for pathway and network analysis for complex studies. The approach used MetaMAPP [28], a network modeling tool that uses KEGG reaction pairs (e.g. standard metabolic pathways) and then adds compounds, which are not on these pathways, by chemical similarity (Tanimoto) index [29].

## Results

Baseline characteristics according to race for the PEAR participants included in this metabolomics study are described in Table 1. GC-TOF data from plasma samples collected before and after 9 weeks of atenolol treatment were analyzed; a total of 544 samples from 272 patients were analyzed. Analysis of plasma on the GC-TOF platform resulted in a total of 157 identified compounds after processing in BinBase. These included amino acids, sugars and sugar alcohols, fatty acids and cholesterol, organic anions, including TCA cycle intermediates, and many other compounds. There were 171 additional compounds in the dataset that were observed and annotated but not identified.

**Table 1:** Baseline Characteristics of Study Participants According to Race (n = 272).

Characteristics	Caucasians (n = 150)	African Americans (n = 122)
Age, years	50.4±9.5	46.9±8.7
Men, n (%)	74 (49.3%)	31 (25.4%)
Weight, kg	88.7±17.3	88.2±18.1
BMI, kg/m^2^	30.5±5.9	31.5±6.5
Waist circumference, cm	97.7±12.7	96.6±13.8
Hip circumference, cm	109.0±10.8	113.5±14.3

Continuous variables are presented as mean ± standard deviation;

Categorical variables are presented as numbers and percentage.

BMI: body mass index.

### Metabolomic Signature of Atenolol Treatment

Study participants on average had expected physiological and metabolic changes over a course of atenolol therapy (Table 2). Systolic and diastolic blood pressure decreased, along with LDL, HDL and plasma renin activity in both Caucasians and African American patients. Glucose, triglycerides and uric acid increased significantly over the course of the 9 weeks. As expected, there were significant difference between Caucasians and African Americans in blood pressure and plasma renin activity change in response to atenolol monotherapy (Table 2).

**Table 2:** Comparison of clinical metabolic parameters for atenolol monotherapy (N = 272) for Caucasians and African Americans.

	Caucasians (n = 150)				African Americans (n = 122)				C vs. AA*
	Baseline	Post-Treatment	Δ	P	Baseline	Post-Treatment	Δ	P	P
**SBP mm Hg**	145.1±9.8	133.6±12.5	−11.48±9.62	<0.0001	144.8±10.7	142.2±13.6	−2.63±10.57	0.007	<0.0001
**DBP mm Hg**	92.4±5.7	82.2±7.4	−10.27±6.77	<0.0001	93.9±6.6	89.9±8.6	−4.02±6.50	<0.0001	<0.0001
**Glucose mg/dl**	91.7±10.6	94.5±10.1	2.73±9.11	0.0003	89.1±10.9	92.0±14.8	2.88±13.47	0.0197	0.33
**LDL mg/dl**	121.7±30.3	118.2±30.7	−3.48±20.35	0.038	122.3±31.0	121.5±33.9	−0.83±17.85	0.61	0.81
**HDL mg/dl**	47.9±12.5	45.2±11.7	−2.73±6.60	<0.0001	55.2±17.6	52.2±16.5	−3.02±7.52	<0.0001	0.91
**Triglyceride mg/dl**	138.1±80.3	164.0±133.4	25.85±79.48	0.0001	93.3±62.7	107.0±65.4	13.74±37.80	0.0001	0.17
**Uric Acid mg/dl**	5.43±1.30	5.71±1.25	0.28±0.60	<0.0001	5.44±1.36	5.70±1.33	0.26±0.71	<0.0001	0.84
**Plasma Renin Activity, ng/ml/h**	1.23±1.36	0.48±0.61	−0.77±1.22	<0.0001	0.61±0.59	0.34±0.43	−0.26±0.56	<0.0001	<0.0001

All variables are presented as mean ± standard deviation.

**Δ:** difference between baseline and post-treatment.

AA: African Americans.

* C vs. AA: p-value for Caucasians vs. African Americans.

**Figure 1:**
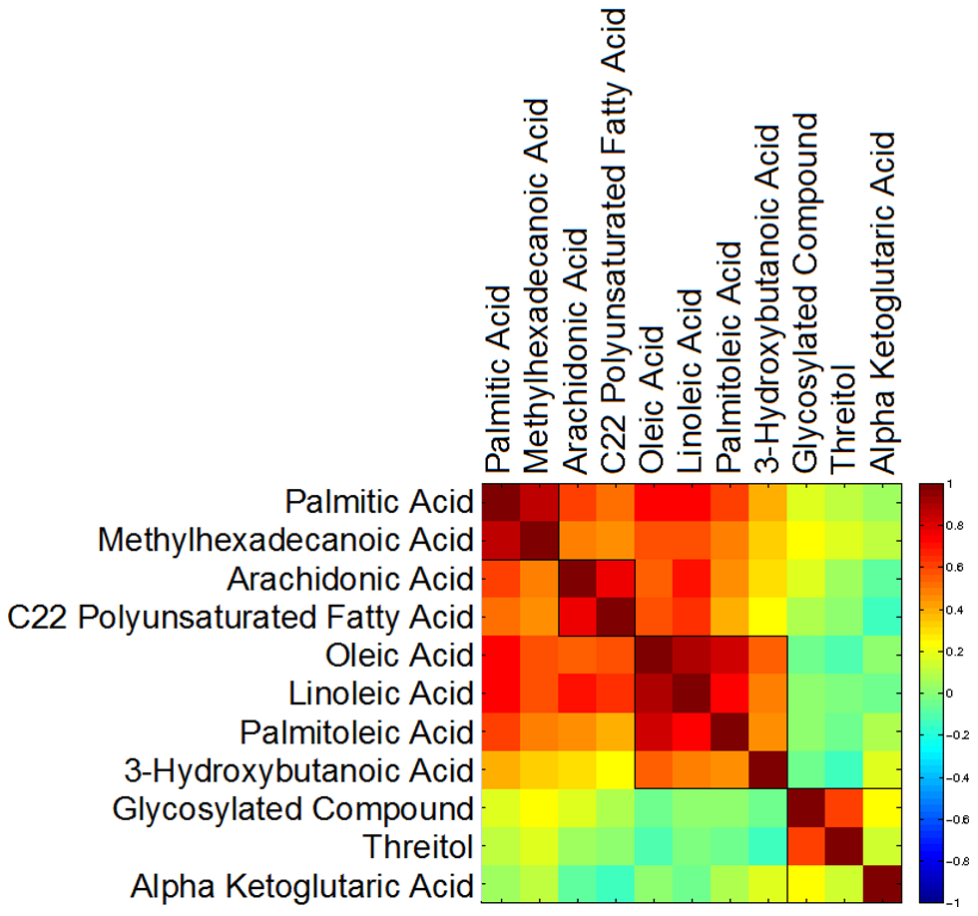
Heat map showing the correlation of the change in a subset of metabolite concentrations with each other. The fatty acids correlate with each other but not with threitol or alpha-ketoglutaric acid.

Seventeen metabolites had a nominally significant change in plasma levels upon atenolol treatment; nine changed significantly in the complete dataset after considering false discovery rate (Table 3) seven of which were fatty acids. Four of these fatty acids, myristic, methylhexadecanoic, palmitic and stearic acids are saturated, whereas palmitoleic and oleic are monounsaturated; arachidonic acid and linoleic are polyunsaturated. These structurally diverse fatty acids decreased in concentration significantly over the treatment period. Many free fatty acids, such as arachidonic acid, oleic, linoleic and palmitic, are non-covalently bound in large quantities to human serum albumin [30]. Fatty acid changes correlated strongly with each other in the total population (Figure 1). Some fatty acids, including the saturated fatty acids lauric (12∶0) and capric (10∶0), caprylic acid (8∶0), pentadecanoic acid (15∶0), and azelaic acid, a saturated dicarboxylic acid, remained unchanged by atenolol treatment across categories. Elaidic acid (18∶1 trans-9), the trans isomer of oleic acid, was not changed.

**Table 3:** Identified compounds that change significantly after atenolol treatment in Caucasians and African Americans (n = 272).

Compound	% change	p-value	Q value
**oleic acid (18∶1 cis-9)**	−17.1	1.18E-07	5.43E-05
**linoleic acid (18∶2 n-6)**	−12.8	1.06E-06	2.44E-04
**palmitoleic acid (16∶1)**	−15.4	2.51E-05	3.85E-03
**palmitic acid (16∶0)**	−9.1	3.42E-05	3.93E-03
**3-hydroxybutanoic acid**	−16.4	4.268E-05	3.93E-03
**arachidonic acid (20∶4 ω-6)**	−9.5	4.33E-04	0.031
**threitol**	6.6	4.67E-04	0.031
**methylhexadecanoic acid (17∶0)**	−7.4	6.16E-04	0.035
**alpha ketoglutaric acid**	9.4	9.26E-04	0.046
**myristic acid (14∶0)**	−4.1	7.95E-03	0.247
**threonine**	−7.1	8.30E-03	0.247
**arabitol**	4.0	8.57E-03	0.247
**stearic acid (18∶0)**	−8.1	0.015	0.295
**dihydroabietic acid**	5.1	0.031	0.474
**glycerol-alpha-phosphate**	−7.1	0.041	0.528
conduritol-beta-epoxide*	6.8	0.044	0.528
**allo-inositol**	5.5	0.049	0.574

Bold line indicates Q<0.05.

The ketone body 3-hydroxybutyrate was significantly reduced upon atenolol treatment in the complete dataset. Two other ketone bodies, acetone and acetoacetate, were not measured in the experiment. The sugar alcohol threitol and the essential amino acid threonine (marginal significance) were also reduced upon atenolol treatment in the combined dataset.

Atenolol treatment did not change the levels of glycerol or glycerol-3-phosphate, either in the combined data or after separating Caucasians and African Americans. Lipids that remained unchanged upon atenolol treatment included alpha and gamma tocopherol and cholesterol.

**Figure 2:**
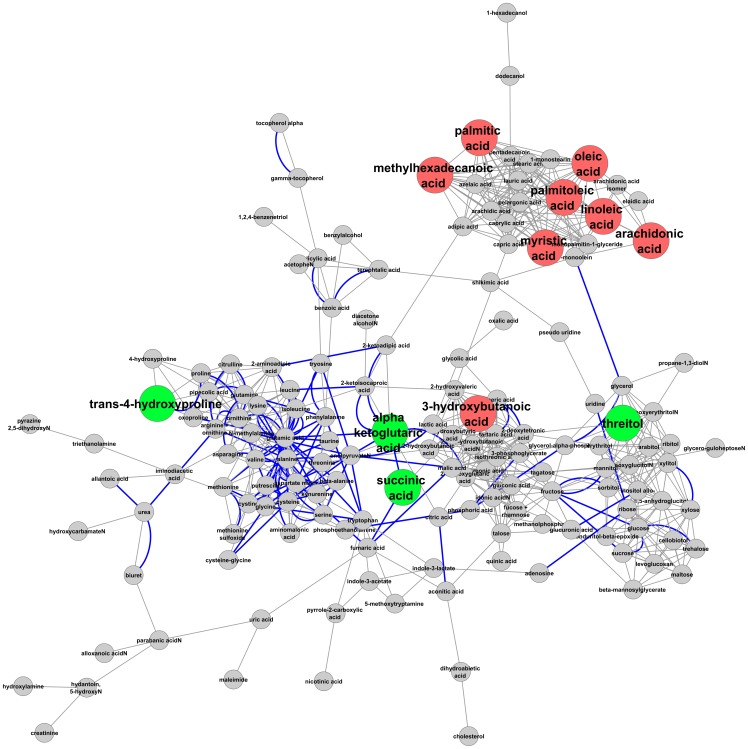
Metabolic network showing changes in Caucasian subjects that occur as a result of atenolol treatment after 9 weeks. Red color indicates that compounds decrease significantly in concentration; green indicates compounds that increase significantly in concentration. Bold blue lines indicate compounds related by a Kegg reaction pair, whereas others are related by structural similarity.

The observed metabolic changes were modeled by constructing a metabolic network using KEGG reaction pairs and structural (Tanimoto) distances, using in part the software MetaMAPP [28] and Cytoscape [24]. This allows for visualization of metabolic changes and the determination of how observed compounds are connected by pathways (Figure 2).

### Metabolomic Signature of Atenolol Treatment Differs in Caucasians and African Americans

There were large differences in the metabolic response to atenolol between Caucasians and African Americans. Many of the changes that appear in the total population (Table 3) are the result of significant changes in the Caucasian population, but not in the African American population (Table 4). Saturated (palmitic), monounsaturated (oleic, palmitoleic) and polyunsaturated (arachidonic, linoleic) free fatty acids all decreased in Caucasians with atenolol treatment after correction for false discovery rate (FDR) with Q-value <0.05. None of these compounds changed significantly in African Americans after correcting for FDR. Similarly, when the data were broken down by race, the reduction in the ketone body 3-hydroxybutyrate was significant in Caucasians with a 33% change but not African American individuals (Table 4).

**Table 4:** Differences in the metabolic response of atenolol treatment between Caucasians and African Americans.

Compound	Caucasians			African-Americans		
	% change	p-value	Q value	% change	p-value	Q value
**oleic acid (18∶1 cis-9)**	−21.1	1.88E-06	0.0009	−9.2	0.007	0.41
**linoleic acid (18∶2 n-6)**	−16.2	4.77E-06	0.0011	−9.6	0.019	0.54
**3-hydroxybutanoic acid**	−33.3	5.03E-05	0.0080	−11.5	0.059	0.67
**palmitic acid (16∶0)**	−9.1	1.12E-04	0.0134	−6.2	0.062	0.67
**palmitoleic acid (16∶1)**	−16.2	3.75E-04	0.0285	−5.4	0.022	0.56
**arachidonic acid(20∶4 ω-6)**	−10.7	4.15E-04	0.0285	−9.9	0.202	0.74
**threitol**	12.2	2.03E-03	0.1218	13.4	0.067	0.67
**methylhexadecanoic acid**	−7.2	3.93E-03	0.2094	−2.4	0.056	0.67
**myristic acid (14∶0)**	−9.5	0.023	0.5583	−2.2	0.152	0.73
**alpha ketoglutaric acid**	10.5	0.033	0.6834	14.5	0.009	0.41
**succinic acid**	9.9	0.037	0.7066	−0.7	0.736	0.85
**trans-4-hydroxyproline**	14.0	0.049	0.7673	10.7	0.848	0.88

The significant (p<0.05) changes in Caucasians are shown with the statistics of the corresponding compound in African Americans for comparison. Q-values are corrected for FDR and the boxed section of the table highlights compounds with Q<0.05.

### Genetic associations

The fatty acid signature of atenolol suggested that lipases might be regulated differently in Caucasians and African Americans and genetic variation might contribute to the differences observed. We therefore tested the association between the top atenolol treatment signature, oleic acid, and the SNPs on the 16 genes encoding lipases on the cardiovascular SNP array. We found that an intronic SNP (rs9652472) on LIPC, the hepatic lipase, was associated with oleic acid response in Caucasians (p = 3.6×10^−4^) but not in African Americans (p = 0.40) after atenolol monotherapy. We also found that an intronic SNP (rs7250148) on PLA2G4C, phospholipase A2 group IVC, was associated with oleic acid change in African Americans (p = 9.6×10^−5^), but not in Caucasians (p = 0.62).

## Discussion

The aim of this study was to evaluate changes in the metabolome induced by treatment with atenolol and determine whether metabolomics provides novel mechanistic insight into racial differences in drug response. Our findings revealed a strong effect of atenolol on fatty acids that differed by race. Indeed, the effects of atenolol were highly significant in Caucasians but absent or minimal in African Americans. Further, we observed a race-dependent genetic association between the changes in oleic acid with SNPs in genes that encode lipases.

### Overall changes and mechanism

We show for the first time a significant difference in the metabolic signature of atenolol treatment between Caucasians and African Americans. Looking at the global changes induced by atenolol (Table 3 and Figure 1), there is a strong signature consisting mainly of plasma free fatty acids, presumably involving either a change in the relative rates of synthesis and/or breakdown. Metabolic pathway analysis, described below, indicates that these fatty acids are not related directly by synthetic pathways (for example β-oxidation). Thus, alteration in a single synthetic pathway could not account for the coordinated changes.

An effect on basal lipolysis would be the most obvious potential mechanism for the major changes in fatty acids observed here: the hydrolysis of triglycerides to free fatty acids and glycerol, followed by further fatty acid breakdown via beta oxidation. Lipolysis is stimulated by hormones, including epinephrine and norepinephrine, and is up-regulated by the β-adrenergic receptors and down-regulated by α2-adrenergic receptors. Epinephrine, a non-specific beta-adrenergic agonist, stimulates lipolysis via the β3-adrenoreceptor (ADRB3). Atenolol specifically, and β-blockers generally, have an effect on plasma lipoprotein metabolism by increasing plasma triglyceride levels and decreasing HDL but not affecting LDL [31]. The effect on triglycerides is smaller with atenolol than propanolol, likely due to the relative β_1_-receptor selectivity of atenolol [31]. Both atenolol and propanolol have been shown to reduce free fatty acid levels [32]. If the reduction in plasma fatty acids were due primarily to general lipolysis, then a corresponding change in both plasma glycerol and glycerol-3-phosphate levels would also be expected, as these are products of triglyceride breakdown. Perhaps the endogenous levels of these compounds are sufficiently large relative to the change in their levels so as to mask the change from observation.

**Figure 3:**
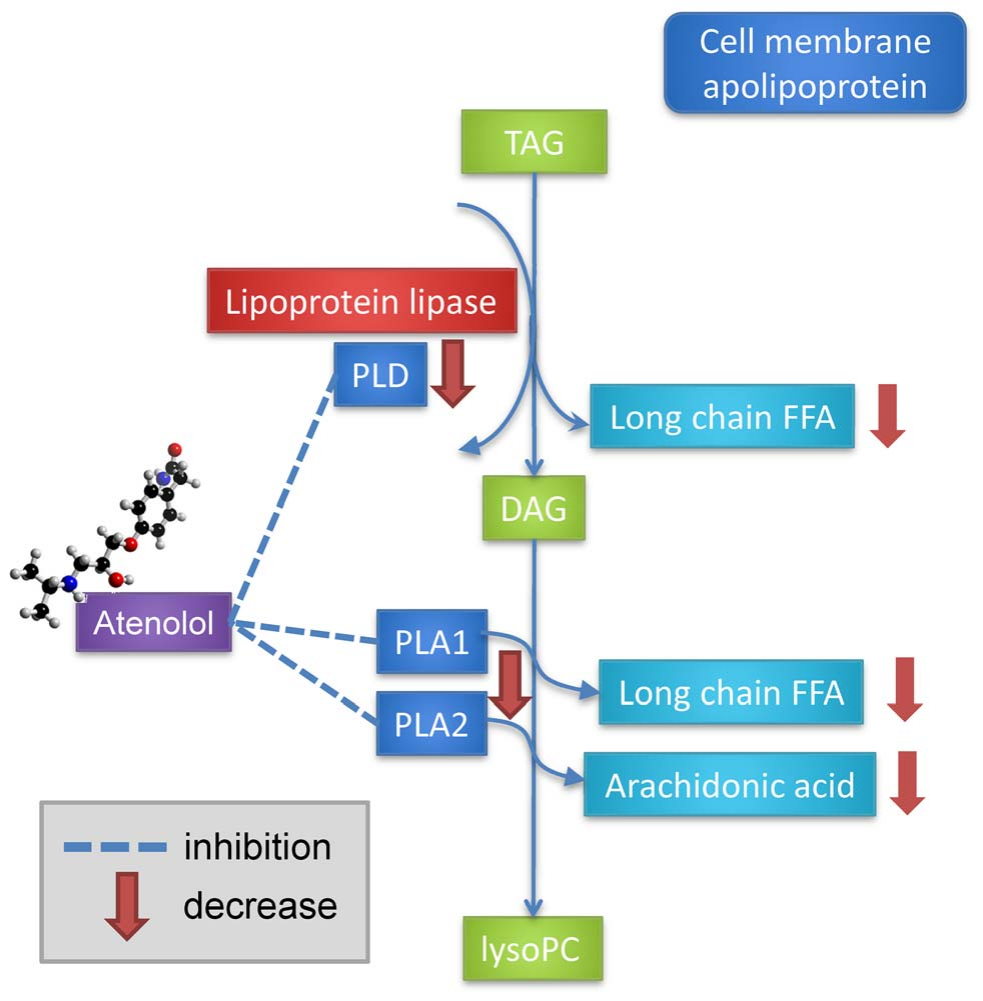
Alternative model of a potential mechanism for atenolol treatment on plasma free fatty acid concentrations.

A second possible mechanism for the fatty acid changes observed may be the direct effect of atenolol on phospholipase activity (Figure 3). This mechanism is conceptually similar to that of changes in lipolysis, although the upstream signaling interaction would be different. There is circumstantial evidence suggesting that β-blockers inhibit lysosomal phospholipase A and C [33]. Atenolol specifically has been found to inhibit lysosomal phospholipase A1, although with less potency than propanolol [34]. This suggests the possibility of a specific mechanism in which atenolol may bind to and inhibit particular phospholipases in plasma or other related tissues (Figure 3). Atenolol has been shown to bind to bee venom phospholipase A2 and form a stable complex. This mechanism also allows for a potential explanation of racial variance, as phospholipase activity has been shown to vary as a function of both sex and race. Lipoprotein-associated phospholipase A2 (Lp-PLA2), for example, was 15% lower in African American individuals compared with Caucasian subjects [35]. Higher concentrations of Lp-PLA2 are associated with increased cardiovascular risk, and cutoffs have been suggested [36].

It has been shown that atenolol-modulated changes in lipids involve more than simply a general adrenergic-mediated alteration in lipolysis rate [37]. In cultured vascular smooth muscle cells, atenolol induced a 61% increase in NAD^+^/NADH ratio and a similar increase in the level of NADH-cytochrome b_5_ reductase [38]. This protein is involved in desaturation and elongation of fatty acids [39], as well as cholesterol biosynthesis [40], suggesting that some of the observed changes could be caused by this enzyme.

The metabolite with the largest percent change was the ketone body 3-hydroxybutyric acid, the concentration of which was reduced by 33.3% in Caucasian individuals (p = 0.000050, Q = 0.0080) upon atenolol treatment but was not significantly altered in African Americans (Table 4). Such a change could result from either decreased production or increased utilization of 3-hydroxybutyric acid, which is produced from acetyl-CoA as a result of ketogenesis, mitochondrial free fatty acid β-oxidation (breakdown), which occurs mostly in the liver [41]. This production typically occurs during periods of low glucose levels, when carbohydrate availability has been reduced. A concentration change may reflect an alteration in the balance of energy usage between carbohydrates and lipids. Note however that glucose undergoes only a slight but significant increase (Table 2), suggesting the 3-hydroxybutyric acid reduction could be the direct result of the reduction in plasma free fatty acids upon atenolol treatment.

### Racial differences in Atenolol treatment

Differences in drug response between racial groups is increasingly recognized as an important aspect of pharmacometabolomics and, more generally, personalized health care [42]–[44]. Atenolol monotherapy is significantly less effective for blood pressure lowering in patients of African origin than for Caucasian patients [45]. While racial differences in plasma renin activity associate with these differences in antihypertensive response, more detailed understanding of the genetic or biochemical factors that underpin these differences in response is needed.

While there is not a clear biochemical mechanism to explain the metabolic differences observed in response to atenolol in Caucasians compared to African Americans, there are differences in lipid metabolism that may relate to specific mechanisms. African Americans have on average higher plasma concentrations of arachidonic acid compared with Caucasians, resulting from genetic variants (SNP rs174537) in the fatty acid desaturase enzyme (FADS gene cluster) that converts linoleic acid to arachidonic acid [46]. For example, experiments on human adipose tissue have shown that the basal lipolysis rate is approximately 50% lower in obese African American women than in Caucasian women and that this may be due to differences in hormone-sensitive lipase enzyme levels [47]. The degree of metabolic inflexibility, which relates to the ability of an individual to switch substrate usage under different metabolic conditions, is different in African American compared with Caucasian women. Caucasians had higher rates of fat oxidation with lower rates of carbohydrate oxidation during a high fat diet in comparison with a low fat diet, whereas African Americans showed no difference [48]. There are known racial differences in the blood pressure response to atenolol between African American and Caucasian patients [49]–[51]. Although physiological differences including both blood pressure and heart rate in the response to atenolol have been characterized between Caucasians and African Americans, metabolic differences have not been previously associated with race.

The racially disparate fatty acid signature induced by atenolol suggested genetic variation may also contribute to the differences observed between Caucasians and African Americans. We therefore tested the association between the top fatty acid signal oleic acid (Table 4) and SNPs on the 16 genes encoding lipases. In Caucasians but not African Americans, the LIPC SNP rs9652472 was associated with oleic acid change (p = 3.6×10^−4^), whereas in African Americans but not Caucasians, the PLA2G4C SNP rs7250148 was associated with oleic acid change (p = 9.6×10^−5^). Thus, the observed differences are explained at least in part by genetic differences that may yield different activities in lipases and corresponding differences in response to atenolol. These findings are consistent with our pharmacometabolomics results indicating that African Americans and Caucasians have distinct signatures in response to atenolol monotherapy.

In summary, we showed that atenolol treatment causes a marked change in plasma fatty acid levels in Caucasians but not African Americans. We also showed that a SNP in the LIPC and PLA2G4C genes were associated with the change in oleic acid in Caucasians and African Americans, respectively. Specific race-dependent changes in other metabolites such as the ketone body 3-hydroxybutanoic acid and TCA cycle intermediate alpha ketoglutaric acid need to be further investigated. Pharmacometabolomics provides powerful tools to understand the mechanistic basis of variation in response to drug therapy. It complements information derived from pharmacogenomics and when combined, enables a systems pharmacology approach to increase our understanding of drug effects.
